# X chromosome aneuploidy in the Alzheimer’s disease brain

**DOI:** 10.1186/1755-8166-7-20

**Published:** 2014-03-06

**Authors:** Yuri B Yurov, Svetlana G Vorsanova, Thomas Liehr, Alexei D Kolotii, Ivan Y Iourov

**Affiliations:** 1 Mental Health Research Center, Russian Academy of Medical Sciences, 117152 Moscow, Russia; 2 Institute of Pediatrics and Children Surgery, Ministry of Health of the Russian Federation, 125412 Moscow, Russia; 3 Moscow City University of Psychology and Education, 127051 Moscow, Russia; 4 Jena University Hospital, Friedrich Schiller University, Institute of Human Genetics, Kollegiengasse 10, D-07743 Jena, Germany; 5 Department of Medical Genetics, Russian Medical Academy of Postgraduate Education, 123995 Moscow, Russia

**Keywords:** Alzheimer’s disease, Aneuploidy, Brain, Chromosome instability, Chromosome X, Molecular cytogenetics, Aging

## Abstract

**Background:**

Although the link between brain aging and Alzheimer’s disease (AD) is a matter of debate, processes hallmarking cellular and tissue senescence have been repeatedly associated with its pathogenesis. Here, we have studied X chromosome aneuploidy (a recognized feature of aged cell populations) in the AD brain.

**Results:**

Extended molecular neurocytogenetic analyses of X chromosome aneuploidy in 10 female AD as well as 10 age and sex matched female control postmortem brain samples was performed by multiprobe/quantitative FISH. Additionally, aneuploidy rate in the brain samples of 5 AD and as 5 age and sex matched control subjects were analyzed by interphase chromosome-specific multicolor banding (ICS-MCB). Totally, 182,500 cells in the AD brain and 182,500 cells in the unaffected brain were analyzed. The mean rate of X chromosome aneuploidy in AD samples was approximately two times higher than in control (control: mean - 1.32%, 95% CI 0.92- 1.71%; AD: mean - 2.79%, 95% CI 1.88-3.69; *P* = 0.013). One AD sample demonstrated mosaic aneuploidy of chromosome X confined to the hippocampus affecting about 10% of cells. ICS-MCB confirmed the presence of X chromosome aneuploidy in the hippocampal tissues of AD brain (control: mean - 1.74%, 95% CI 1.38- 2.10%; AD: mean - 4.92%, 95% CI 1.14-8.71; *P* < 0.001).

**Conclusions:**

Addressing X chromosome number variation in the brain, we observed that somatically acquired (post-zygotic) aneuploidy causes large-scale genomic alterations in neural cells of AD patients and, therefore, can be involved in pathogenesis of this common neurodegenerative disorder. In the context of debates about possible interplay between brain aging and AD neurodegeneration, our findings suggest that X chromosome aneuploidy can contribute to both processes. To this end we conclude that mosaic aneuploidy in the brain is a new non-heritable genetic factor predisposing to AD.

## Background

Alzheimer’s disease (AD) represents one of the most common age-related neurodegenerative disorders with a strong genetic basis. Currently, a widely accepted model of AD genetics proposes that this devastating pathology is associated with several genetic defects including single-gene mutations, risk-enhancing single nucleotide polymorphisms (SNP) and copy number variations (CNV), genetic instabilities (at chromosomal or sub-chromosomal level), and a complex pattern of genetic-environmental interactions [1-3]. Parallelly, a debate questioning the existence of interplay between brain aging and AD pathology does take place in order to critically address the contribution of cell senescence and related phenomena to AD neurodegeneration [4,5]. Nonetheless, there is a consensus about AD genetic background, which is hypothesized, among others, to result in alteration to neuronal cell cycle.

A series of studies has provided indirect evidences that abnormal behavior of chromosome X during the cell division is observed in AD patients [6-8]. Furthermore, some late-onset AD cases were linked to chromosome X [9]. On the other hand, mosaic numerical chromosome imbalances (somatic aneuploidy) have been repeatedly observed in the AD and unaffected (aged) human brain and are now considered as an integrated part of the pathogenic cascade mediating progressive neurodegeneration in this devastating neurological disease [10-20]. Since X chromosome loss is one of the most prominent hallmarks of aging in human females [21] and an association between aging and aneuploidy in the murine brain has been shown [22], it is attractive to test whether X chromosome aneuploidy can be an element of AD pathogenic cascade at least in cases of neurodegeneration in the diseased brain of females. Here, we used a set of molecular cytogenetic techniques [23-25] providing high-resolution analysis of interphase chromosomes to detect genome variations manifesting at chromosomal level in small cell populations for a molecular neurocytogenetic analysis of the AD brain and control samples.

## Results

**Figure 1:**
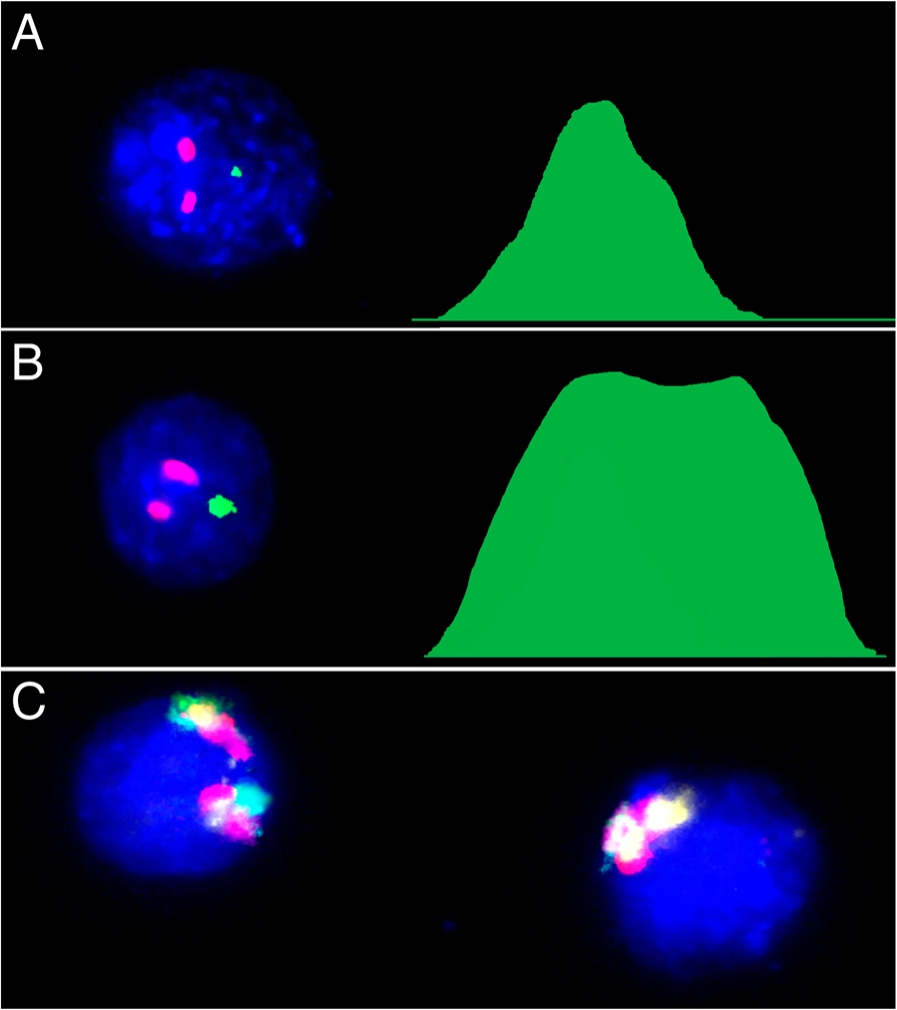
**Molecular neurocytogenetic analyses of the AD brain. (A)** multiprobe (two-probe) and quantitative FISH using DNA probes for chromosomes 1 (two red signals/D1Z1) and X (one green signal DXZ1/relative intensity is 2120 pixels) demonstrating true X chromosome monosomy; **(B)** multiprobe (two-probe) and quantitative FISH using DNA probes for chromosomes 1 (two red signals/D1Z1) and X (one green signal DXZ1/relative intensity is 4800 pixels) demonstrating overlapping of two X chromosome signals, but not a chromosome loss; **(C)** ICS-MCB with a probe set for chromosome X showing one nucleus bearing two chromosomes X and another nucleus bearing single chromosome X.

Using multiprobe/quantitative FISH and interphase chromosome-specific multicolor banding (ICS-MCB) (Figure 1), we have assessed the rate of aneuploidy involving different chromosomes in postmortem brain tissues of 10 control and 10 AD patients analyzed in a double-blinded study. Chromosome enumeration probes for six different autosomes (1, 7, 11, 16, 17, and 18) and chromosome X were applied. 2000 cells were scored for each DNA probe/brain tissue sample. Following combination of chromosome-enumeration probes were used: chromosomes 1, X, Y (as internal control for the sex of subjects analyzed/stringency of hybridization); chromosomes 1, 7, 11 and chromosomes 16, 17 and Y. In total, scoring more than 140,000 cells in control samples and comparable amount of cells (140,000) in the AD brain has demonstrated aneuploidy rates (losses + gains) to vary in a wide range between chromosomes and individuals (Table 1). In average, aneuploid cell populations demonstrated chromosome gains in 10-20% of cells, whereas 80-90% of cells exhibited chromosome losses. Cells affected by multiple aneuploidies were not detected. Interphase nucleus morphology and numbers have not significantly differed between AD and control samples. In control samples, the mean frequency of autosomal aneuploidy ranged between 0.51 and 0.82 with an “autosomal” mean determined as 0.66 (95% CI, 0.57-0.7%). The mean of X chromosome aneuploidy rates in controls was 1.32% (95% CI, 0.92-1.71%). In the AD brain, the mean autosomal aneuploidy frequency was in a range of 0.86-1.22% and “autosomal” mean was 0.93% (95% CI, 0.78-1.07%) The mean frequency of X chromosome aneuploidy rates was 2.78% (95% CI, 1.88-3.68). We have compared the frequency of aneuploidy between each homologous chromosome pair by scoring 20000 cells in control and 20000 cells in AD groups (Table 1) using nonparametric statistical tests (Mann-Whitney *U* test for independent groups). Insignificant interindividual differences between autosomal aneuploidy rates were obtained in controls and AD (*P* value ranged between 0.053 and 0.733). The increase of X chromosome aneuploidy rates in the AD cerebrum was significantly (*P* value = 0.013).

**Table 1:** Aneuploidy rates in the AD and normal prefrontal cortex

**Controls**	**Chromosomes/percent aneuploidy (losses + gains)**						
**No (age, years)**	**1**	**7**	**11**	**16**	**17**	**18**	**X**
Control							
AK1(76)	0.8	0.9	0.3	0.4	1.2	1.4	0.9
AK2 (78)	0.9	0.6	1.5	0.6	0.5	1.2	1.6
AK3 (80)	0.5	1.0	0.9	0.6	0.8	0.8	0.7
AK4 (82)	0.3	1.4	0.6	1.6	0.7	1.3	1.2
AK5 (79)	0.6	0.9	0.8	0.8	0.8	1.0	1.4
AK6 (69)	0.2	0.4	0.9	0.5	0.6	0.4	0.8
AK7 (71)	0.4	0.6	0.3	0.4	0.3	0.5	1.2
AK8 (70)	0.3	0.2	0/5	0.6	0.5	0.2	2.6
AK9 (82)	0.6	0.4	0.3	0.7	0.5	0.3	1.2
AK10 (79)	0.5	0.3	0.7	0.2	0.4	1.1	1.6
The mean	0.51	0.67	0.68	0.64	0.63	0.82	1.32
(95% CI)	(0.35-0.67)	(0.40-0.94)	(0.41-0.95)	(0.37-0.91)	(0.44-0.81)	(0.50-1.14)	(0.92-1.71)
AD							
AD1 (72)	0.5	1.3	1.7	0.3	0.8	0.5	1.0
AD2 (78)	0.2	1.2	0.7	0.9	0.7	0.3	1.0
AD3 (80)	0.4	2.7	0.5	0.8	1.4	1.4	1.6
AD4 (80)	1.0	0.9	0.9	2.3	0.9	1.1	2.3
AD5 (88)	1.3	2.3	1.6	2.5	1.1	1.6	3.9
AD6(82)	0.9	0.8	0.6	0.7	0.4	0.5	4
AD7 (76)	1.1	0.4	0.6	0.4	0.4	0.3	3.3
AD8 (81)	0.6	0.7	0.4	0.5	0.5	1.4	2.7
AD9 (69)	0.7	0.9	0.8	0.4	1.2	0.6	4.3
AD10 (72)	1.2	1.0	1.1	0.5	1.4	0.9	3.7
The mean	0.79	1.22	0.89	1.53	0.88	0.86	2.78
(95% CI)	(0.53-1.05)	(0.70-1.74)	(0.57-1.21)	(0.72-2.34)	(0.60-1.15)	(0.51-1.21)	(1.88-3.68)
*P* value (Mann-Whitney *U* test)	0.075	0.053	0.273	0.597	0.162	0.733	0.013
Shapiro-Wilk’s W test for normality (outliers excluded)	0.838	2.676	0.105	0.731	0.32	0.2	0.226
*P* value (*t* test)	0.054	0.047	0.27	0.313	0.106	0.85	0.003

(2000 cells per chromosome were scored for each sample; a *P* value of less than 0.05 was considered as significant; Mann-Whitney *U*-test for independent groups).

To get further insight into the contribution of somatic gonosomal aneuploidy to the AD brain pathology, we have compared X chromosome aneuploidy rates in different brain areas (hippocampus and cerebrum) of 5 AD patients (22500 cells) and 5 controls (22500 cells). ICS-MCB and multiprobe/quantitative FISH have shown a dramatic increase of X chromosome aneuploidy rates in hippocampal cells of AD patients. The mean rate of chromosome X aneuploidy was 1.74% (95% CI, 1.38-2.10%) in controls and 4.92% (95% CI, 1.14-8.71%) in AD (*P* value <0.001).

**Figure 2:**
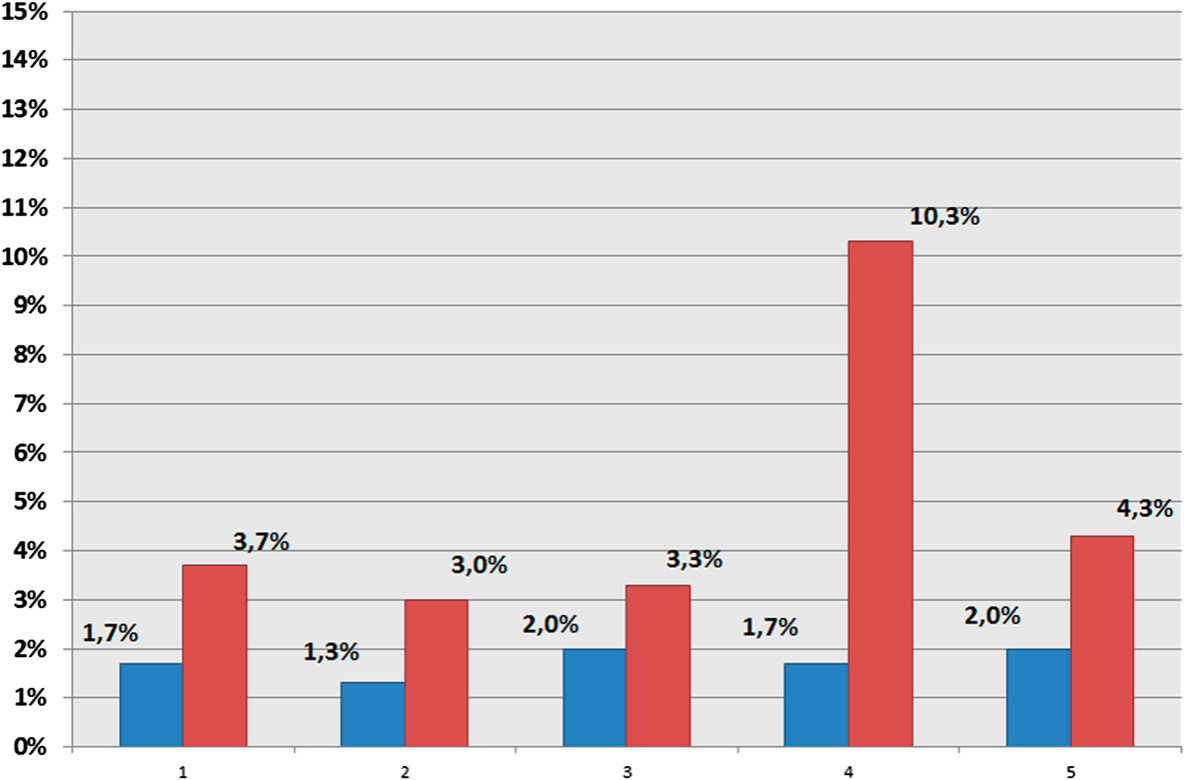
X chromosome aneuploidy in the hippocampus of the AD brain and controls analyzed by ICS-MCB (AD: n = 5, red bars; control: n = 5, blue bars); AD: mean 4.92%, 95% CI 1.14-8.71; control: mean 1.74%, 95% CI 1.38-2.1; X axis: number of samples’ pairs: AD patient—age-matched control; Y axis: rates of aneuploidy (given in %).

X chromosome aneuploidy levels detected in 5 AD patients were verified in the prefrontal cortex (the second brain tissue affected by neurodegeneration). The mean rate of chromosome X aneuploidy in the cerebrum was 1.16% (95% CI, 0.56-1.76%) in controls and 2.84% (95% CI, 1.78-3.90%) in AD (*P* value = 0.009). Surprisingly, one sample derived from the hippocampus of an AD patient (Figure 2 A, B) was found to exhibit low-level somatic chromosomal mosaicism (about 10% of cells were affected by X chromosome aneuploidy) almost exclusively confined to the hippocampus (Figure 2B). To our knowledge, cases of AD demonstrating brain-specific (brain-area-specific) chromosomal mosaicism involving chromosome X have been never reported.

## Discussion

The hypothesis suggesting aneuploidy (trisomy of chromosome 21) to be involved in AD pathogenesis has long been explored through addressing mitotic tissues [26-28]. However, until recently, no consensus was reached regarding the true occurrence of somatic aneuploidy in the AD brain [12-20,27,28]. The intercellular genomic variations affecting the AD brain were assumed to be limited to chromosome 21 aneuploidy because of neurological parallels between AD and Down syndrome [12,26,29,30]. This assumption was further supported by reports showing that molecular dysfunctions in AD neural cells are likely to predispose to formation of chromosome 21-specific aneuploidy [19,29,30]. Alternatively, brain-specific chromosome instability manifesting as aneuploidy involving different chromosomes was detected in AD [11-15,17,18], but low-level mosaic chromosome 21-specific aneuploidy was found to feature the AD brain [12]. Thus, chromosomal (genomic instability) seems to mediate neurodegeneration in AD regardless of chromosomes involved in aneuploidy [31]. Interestingly, mosaic aneuploidy affecting the human brain is probably a general genetic mechanism for neurodegenerative and psychiatric diseases [10,12,20,32-37]. Only rarely, however, X chromosome aneuploidy is observed in the diseased brain of AD patients. Since genetic brain diseases (apart from AD and ataxia telangiectasia) studied according to molecular neurocytogenetic paradigm are not usually associated with aging (i.e. autism and schizophrenia [32-37]), one can speculate that such a genetic hallmark of human aging as X chromosome aneuploidy [15,21,34] should be observed as in the healthy aged brain as in the diseased brain. Furthermore, several evaluations of X chromosome aneuploidy in the AD brain have yielded contradictory results [12,17,38]. Nevertheless, the analysis of X chromosome number variations was not the focus of these studies. The present report demonstrates that X chromosome aneuploidy affects neural cells more significantly in the AD brain when compared to unaffected individuals. Therefore, it is to address an important issue concerning the origins and pathogenic value of mosaic aneuploidy in the AD brain.

The human brain is mainly populated by postmitotic cells. Since somatic aneuploidy is likely to occur during mitotic division, it is difficult to propose a universal mechanism for an increase of aneuploidy rates in the aging human brain. Though, there are a couple of possible solutions for this paradox:

(i) chromosome instability in the human developmental central nervous system is a source for aneuploid cells in postnatal brain [39-41]; failed clearance of abnormal cells during prenatal brain development has to lead to persistence of abnormal neural cell populations; this mechanism seems to be appropriate not only for brain diseases mediated by somatic genome variations in neural cells, but also for somatic mosaicism, as a whole [42]; the persistence of abnormal cells is hypothesized to arise from risk-enhancing SNP or CNV frequently found in AD patients affecting genome stability and programmed cell death pathways [3]. Therefore, mosaic (postzygotic) aneuploidy acquiring during early brain development may be considered as a new non-heritable genetic factor predisposing to late onset AD.

(ii) adult neurogenesis (gliogenesis) during ontogeny can also be considered as a mechanism explaining accumulation of aneuploid cells in the aging brain. It was proposed that neural cell cycle is prone to errors (i.e. abortive cell cycle due to reentering of quiescent neurons into the cell cycle and replication stress) [43] that leads to genome/chromosome instability similar to cancer manifesting as aneuploidy or chromosomal double-strand breaks, resulting, however, in neurodegeneration [44]; the idea is also supported by observations that ectopic cell cycle re-entry of neurons is an element of AD pathogenic cascade [3,4,18,43,45-47].

In the light of the AD cell cycle theory, the presence of X chromosome aneuploidy in the diseased brain does not seem to be unexplainable. Hence, this genetic marker evidence for pathological brain aging as susceptibility factor for AD. Moreover, a case exhibiting appreciable increase of X chromosome aneuploidy confined to the hippocampus (a brain area that is severely affected in AD [1,4,5]) allows speculations about possible predisposition of females affected by low-level mosaic X chromosome aneuploidy to AD. In total, X chromosome aneuploidy appears to play a role in both brain aging and neurodegeneration, whereas aging-related processes are unlikely to cause aneuploidy in the AD brain.

## Conclusions

Molecular neurocytogenetic analysis has shown that X chromosome aneuploidy is a cause of large-scale genomic variation in neural cells of AD patients and unaffected controls. The AD brain demonstrates a two-fold increase of X chromosome aneuploidy rates in neural cells of the hippocampus and cerebrum, which are the brain areas dramatically affected by neurodegeneration. Brain-specific X chromosome aneuploidy can be considered an element of AD pathogenesis, bearing in mind that it results from a series of molecular and cellular events more critical for neurodegeneration. Finally, X chromosome aneuploidy is likely to contribute to both pathological and natural brain aging in humans.

## Methods

### Tissue collection and sample preparation

Postmortem brain tissues (10 AD female and 10 age- and sex matched samples) were obtained from the Postmortem Brain Tissue Bank of Mental Health Research Center, Russian Academy of Medical Sciences. The review board of Ethical Committee at Russian Academy of Medical Sciences (Moscow, Russia) approved all the research procedures. Informed consents or waivers of consents were not required as all case subjects were deceased and anonymously diagnosed. AD diagnosis was based on the results of postmortem evaluations. All control cases were free of mental illness and malignant brain pathology. Death causes were not associated with brain diseases, injuries or conspicuous morphological abnormalities of the brain. In 5 AD cases and 5 controls, hippocampal and cerebral tissues were also acquired. The processing of frozen post-mortem tissues for molecular cytogenetic analyses was performed as described earlier in details [48].

### Multiprobe FISH

Multiprobe FISH with chromosome-enumeration DNA probes for chromosomes 1 (D1Z1/Cy3-labeled), 7 (D7Z1/FITC-labeled), 11 (D11Z1/FITC-labeled), 16 (D16Z2/FITC-labeled), 17 (D17Z1/Cy3-labeled), 18 (D18Z1/Cy3-labelled) and X (DXZ1/FITC-labeled) were performed according to previous protocols [12,32,33,36,39-41,44,49].

### Qantitative FISH

Nuclei showing single signals were digitalized and evaluated by an original quantitative FISH technique described previously [50]. Similarly, a quantification of ICS-MCB signals was done for differing between chromosome overlapping and monosomies [51].

### ICS-MCB

ICS-MCB patterns were generated with a set of human microdissection probes for chromosome X on interphase nuclei isolated from the human brain following previously developed protocols [41,51,52]. This method represents a three-to-five-color FISH-based approach producing a reproducible fluorochrome profile along interphase chromosomal axes for determination of the number and structure of interphase chromosomes [51,52].

### Data and image analysis

The whole intermixed population of nuclei consisted of both neural (different types of neurons and glia) and non-neural cells have been analyzed. As aneuploidy was defined to represent an exceedingly rare event in brain cell populations >2000 nuclei per chromosome per sample were analyzed. No fewer than 500 interphase nuclei per chromosome per sample were analyzed using ICS-MCB. The procedure of cell scoring and image analysis including digitalization of wild-type ICS-MCB images for differentiation between “overlapped” chromosomes and true monosomies has been previously described step-by-step in detail [12,36,40,41,44,49,51,52].

### Statistics

Mean frequency and 95% confidence interval for aneuplody rates were determined. To compare stochastic (background) aneuploidy in two groups (AD and age-matched controls), nonparametric statistics (Mann-Whitney *U*-test for independent groups) was used. *P* values less than 0.05 were considered as significant. Shapiro-Wilk’s W test was used for descriptive statistical analysis of distributions for normality in two ways: with outliers and without outliers (distributions were considered normal at P > 0.050). An independent sample *T*-test was used in cases of normally distributed parameters after exclusion of outliers. P values less than 0.05 was considered as significant.

## Abbreviations

AD: Alzheimer’s disease; CNV: Copy number variations; FITC: Fluorescein-isothiocyanate; FISH: Fluorescence in situ hybridization; ICS-MCB: Interphase chromosome-specific multicolor banding; SNP: Single nucleotide polymorphisms.

## Competing interests

The authors declare that they have no competing interests.

## Authors’ contributions

YBY, SGV, TL, and IYI conceived the research, designed the study, and wrote the manuscript. YBY, ADK and IYI performed the experiments. YBY, SGV, TL, and IYI conceived the project and obtained the funding. YBY and TL contributed important reagents. All authors have read and approved the final manuscript.
